# The Involvement of Mig1 from *Xanthophyllomyces dendrorhous* in Catabolic Repression: An Active Mechanism Contributing to the Regulation of Carotenoid Production

**DOI:** 10.1371/journal.pone.0162838

**Published:** 2016-09-13

**Authors:** Jennifer Alcaíno, Natalia Bravo, Pamela Córdova, Andrés E. Marcoleta, Gabriela Contreras, Salvador Barahona, Dionisia Sepúlveda, María Fernández-Lobato, Marcelo Baeza, Víctor Cifuentes

**Affiliations:** 1 Laboratorio de Genética, Departamento de Ciencias Ecológicas, Facultad de Ciencias, Universidad de Chile, Santiago, Chile; 2 Departamento de Biología, Facultad de Ciencias, Universidad de Chile, Santiago, Chile; 3 Centro de Biología Molecular Severo Ochoa, Departamento de Biología Molecular (UAM-CSIC), Universidad Autónoma, Madrid, Cantoblanco, España; University of Szeged, HUNGARY

## Abstract

The red yeast *X*. *dendrorhous* is one of the few natural sources of astaxanthin, a carotenoid used in aquaculture for salmonid fish pigmentation and in the cosmetic and pharmaceutical industries for its antioxidant properties. Genetic control of carotenogenesis is well characterized in this yeast; however, little is known about the regulation of the carotenogenesis process. Several lines of evidence have suggested that carotenogenesis is regulated by catabolic repression, and the aim of this work was to identify and functionally characterize the *X*. *dendrorhous MIG1* gene encoding the catabolic repressor Mig1, which mediates transcriptional glucose-dependent repression in other yeasts and fungi. The identified gene encodes a protein of 863 amino acids that demonstrates the characteristic conserved features of Mig1 proteins, and binds *in vitro* to DNA fragments containing Mig1 boxes. Gene functionality was demonstrated by heterologous complementation in a *S*. *cerevisiae mig1*^-^ strain; several aspects of catabolic repression were restored by the *X*. *dendrorhous MIG1* gene. Additionally, a *X*. *dendrorhous mig1*^-^ mutant was constructed and demonstrated a higher carotenoid content than the wild-type strain. Most important, the *mig1*^*-*^ mutation alleviated the glucose-mediated repression of carotenogenesis in *X*. *dendrorhous*: the addition of glucose to *mig1*^-^ and wild-type cultures promoted the growth of both strains, but carotenoid synthesis was observed only in the mutant strain. Transcriptomic and RT-qPCR analyses revealed that several genes were differentially expressed between *X*. *dendrorhous mig1*^-^ and the wild-type strain when cultured with glucose as the sole carbon source. The results obtained in this study demonstrate that catabolic repression in *X*. *dendrorhous* is an active process in which the identified *MIG1* gene product plays a central role in the regulation of several biological processes, including carotenogenesis.

## Introduction

Most microorganisms can utilize a variety of substrates as carbon sources for growth; however, hierarchical mechanisms have been developed to preferentially utilize glucose over other carbon sources when this sugar is available. In general, the presence of glucose in the culture medium leads to rapid and dramatic repression of the expression of a large number of genes, including altered expression patterns of genes involved in the metabolism of alternative carbon sources. This phenomenon is termed catabolic repression, and it has been extensively studied in the model yeast *Saccharomyces cerevisiae* [1].

Although glucose-dependent regulation can occur at different steps during gene expression, major effects are observed at the transcriptional level, causing a decrease in the transcript abundance of target genes due to the action of negative regulators that enter the nucleus and bind to DNA when glucose is present in the medium [1–3]. Among them, the Mig1 protein (encoded by *MIG1*), which has been described in *S*. *cerevisiae* [4], recognizes and binds to specific DNA sequences called “Mig1 boxes” in the promoter regions of several genes repressed by glucose, including the *SUC* (sucrose), *GAL* (galactose) and *MAL* (maltose) gene families, among others [5–8]. The subcellular localization of Mig1 is regulated by phosphorylation. In the absence of glucose, Mig1 is phosphorylated and localizes to the cytoplasm. However, in the presence of glucose, this sugar is sensed at the cell membrane [9,10], activating an intracellular signaling cascade that leads to the activation of the Reg1-Glc7-phosphatase complex, which dephosphorylates Mig1 [11,12]. In this state, Mig1 migrates to the nucleus where it binds to the promoters of target genes [1,13] and recruits a co-repressor complex formed by the proteins Tup1 and Cyc8, which exerts transcriptional repression functions [8,14,15]. When glucose levels decrease, Mig1 is phosphorylated by the Snf1 kinase complex, causing it to lose its interaction with the Cyc8-Tup1 complex and be transported to the cytoplasm, releasing target gene repression [16].

**Fig 1:**
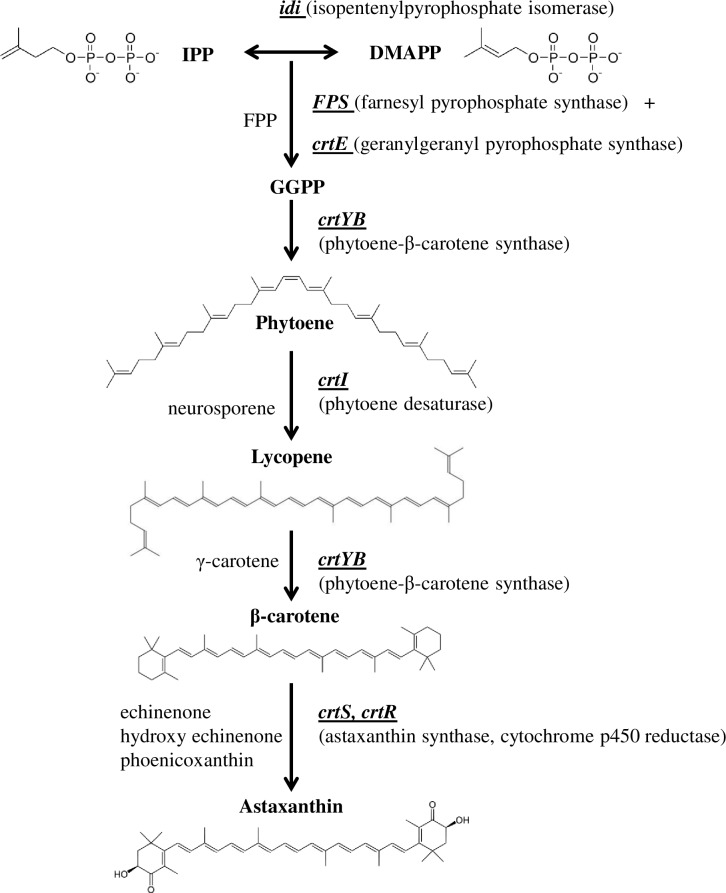
Synthesis of astaxanthin in *X*. *dendrorhous*. The seven structural genes controlling each step of carotenoid synthesis from IPP in *X*. *dendrorhous* are shown. The encoded enzymes are in parenthesis, and intermediary carotenoids are listed to the left of the pathway. The chemical structures of the precursors and of the majors’ carotenoids produced are represented. Abbreviations: IPP: isopentenyl pyrophosphate, DMAPP: dimethylallyl pyrophosphate, FPP: farnesyl pyrophosphate, GGPP: geranylgeranyl pyrophosphate.

Our group has studied the genetic control and regulation of the carotenogenic pathway in the basidiomycete yeast *Xanthophyllomyces dendrorhous* (formerly *Phaffia rhodozyma*). Several lines of evidence suggest that this pathway might be regulated by catabolic repression. *X*. *dendrorhous* possesses the unique ability to produce carotenoids from the fermentation of various sugars, of which astaxanthin is the most abundant [17]. Astaxanthin has many commercial applications: it is currently used in the pharmaceutical and cosmetic industries due to its extremely potent antioxidant properties, and it is widely used in aquaculture to induce the characteristic pigmentation of salmon; as such, it is a significant factor in production costs [18]. In *X*. *dendrorhous*, the synthesis of astaxanthin (Fig 1) derives from the mevalonate pathway, which produces isopentenyl pyrophosphate (IPP), the general precursor of isoprenoids. In carotenoid biosynthesis, IPP is isomerized to dimethylallyl pyrophosphate (DMAPP) by IPP isomerase, which is encoded by the *idi* gene [19]. Then, three IPP molecules are sequentially added to DMAPP by the prenyl transferase farnesyl pyrophosphate synthase (encoded by *FPS*) and geranylgeranyl pyrophosphate (GGPP) synthase (*crtE* gene), giving rise to geranylgeranyl pyrophosphate (GGPP) [20]. Next, two molecules of GGPP are condensed by the phytoene-β-carotene synthase (PBS, encoded by *crtYB*); this is the first exclusively carotenogenic step that produces phytoene, which is colorless [21]. Phytoene undergoes four desaturation reactions by phytoene desaturase (*crtI* gene) to form lycopene (which is red) [22]. The latter is converted into β-carotene (yellow) by the lycopene-cyclase activity of the PBS enzyme. Finally, hydroxyl and keto groups are added to β-carotene, generating astaxanthin (red-orange) as the final product of this pathway. This last step is carried out by astaxanthin synthase (*crtS* gene), which is a cytochrome P450 monooxygenase [23,24]. Thus, this step also involves a cytochrome P450 reductase encoded by the *crtR* gene [25], which supplies the electrons necessary for astaxanthin synthase activity.

Although much is known about the genetic determinants involved in carotenogenesis in *X*. *dendrorhous*, knowledge of its regulation is scarce. It has been observed that the initiation of carotenogenesis during yeast growth depends on the carbon source present in the culture medium. For example, when wild-type *X*. *dendrorhous* is cultured with succinate, a nonfermentable carbon source, carotenogenesis starts when culture begins; however, when glucose is used as the sole carbon source, carotenoid production is lower than that observed with succinate, and most importantly, carotenogenesis only begins by the end of the exponential phase of growth when glucose is exhausted in the culture medium [26], suggesting that this sugar represses carotenogenesis. In line with these observations, when the *X*. *dendrorhous* astaxanthin synthase encoding gene was first described, putative CreA binding motifs (the Mig1 homolog in *Aspergillus nidulans* [27]) were found in its promoter [23]. Similar binding sites have also been found in the promoter regions of the carotenogenic genes *crtYB* and *crtI*, positioned at an equivalent distance from the start codon of each gene [26]. Furthermore, the transcripts of the carotenogenic genes *crtI*, *crtYB* and *crtS* reach maximum levels during the late exponential phase of growth, a time point that coincides with the induction of carotenogenesis and the depletion of glucose in the medium [26]. Moreover, the addition of glucose to the culture medium inhibits the synthesis of carotenoids, coinciding with a decrease in *crtI*, *crtYB* and *crtS* mRNA levels [28].

This background strongly suggests that a catabolic repression mechanism could be responsible for the repression of the carotenogenic genes in *X*. *dendrorhous* in the presence of glucose. Additionally, as in other yeasts, the expression of genes such as *INV* (invertase) and *grg2* (glucose-repressible gene 2) is repressed by glucose in *X*. *dendrorhous* [28,29], supporting the hypothesis that catabolic repression regulates the expression of genes in this yeast and that carotenogenesis could potentially be one of its targets. Although catabolic repression is one of the most conserved regulatory mechanisms among eukaryotes, components that mediate glucose repressive effects in *X*. *dendrorhous* are still unknown. The aim of this work was to identify and characterize the gene encoding *X*. *dendrorhous* Mig1, as this protein has a protagonist role in the catabolic repression process.

## Materials and Methods

### Strains and culture conditions

The strains that were used and constructed in this work are described in Table 1.

**Table 1:** Strains and plasmids used in this work.

Strain/plasmid	Description	Reference or source
**- STRAINS:**		
***E*. *coli*:**		
DH5α	Amp^S^. Used for plasmid propagation.	[30]
BL21 (DE3) PLysS	Kan^S^. Used for gene expression. F-, dcm ompT hsdS (rb- mb-) gal λ (DE3) [pLysSCamr].	Novagen (Merck Millipore, Billerica, MA USA).
***S*. *cerevisiae*:**		
S288c	Haploid wild-type strain	ATCC 204508
YGL035C BY4743 (*Scmig1*^-^)	Haploid *mig1*^-^ mutant strain derived from S288c. Genotype: *MATa his3delta1 leu2delta0 met15delta0 ura3delta0 deltaMIG1*.	[31] ATCC 4034403
*Scmig1*^*-*^(YXd)	*Scmig1*^-^ strain carrying YEpNP-MIG1 (vector to express the *X*. *dendrorhous MIG1* gene).	This work
*Scmig1*^*-*^(Y-)	*Scmig1*^-^ strain carrying YEpAct4 (empty vector).	This work
***X*. *dendrorhous*:**		
UCD 67–385	Diploid wild-type strain (Hyg^S^ and Zeo^S^).	[32]; ATCC 24230
*Xdmig1*^*-/-*^	Hyg^R^ homozygous mutant (*mig1*^*-*^/*mig1*^*-*^*)* derived from UCD 67–385 obtained after transformation and DRM (Double Recombinant method) application.	This work
**- PLASMIDS:**		
pBluescript II SK-	Cloning vector. (AmpR, ColE1 ori, white/blue colony selection).	Agilent Technologies (Santa Clara, CA, USA)
pMN-*hph*	pBluescript II SK containing a 1.8-kb module that confers resistance to hygromycin B at the *Eco*RV site.	[33]
pXdcMIG1	pBluescript II SK containing the *X*. *dendrorhous MIG1* cDNA (2,592 bp) at the *Eco*RV site. The complete *MIG1* cDNA was obtained by performing overlap-extension PCR with two RT-PCR fragments amplified using the following primer pairs: Mig1cDNA + Mig1cDNARV2 (approximately 1,100 bp), and Mig1_Fw1480 + Mig1cDNA_Stop (approximately 1,600 bp).	This work
YEpAct4	*S*. *cerevisiae* expression vector. It contains the *S*. *cerevisiae* Act4 constitutive promoter for expression and the *LEU2* gene for selection.	[34]
YEp-NP	*S*. *cerevisiae* expression vector modified from YEpAct4, with a *Xba*I restriction site between the *S*. *cerevisiae* Act4 constitutive promoter and the TDH3t terminator.	[35]
YEpNP-MIG1	*S*. *cerevisiae* expression vector derived from YEp-NP containing the *X*. *dendrorhous MIG1* cDNA at the *Xba*I site.	This work
pET-TEV	Modified from pET28a (+) (Novagen, Merck Millipore, Billerica, MA USA), the thrombin cleavage site was replaced with the tobacco etch virus (TEV) cleavage site.	[36]
pET-Mig1cDNA	*E*. *coli* expression vector derived from pET-TEV containing the *X*. *dendrorhous MIG1* cDNA between the *No*tI and *Hin*dIII sites.	This work

Amp^S^: sensitive to ampicillin; Hyg^S^: sensitive to hygromycin B; Hyg^R^: resistant to hygromycin B. ATCC: American Type Culture Collection. AmpR: ampicillin resistance; ColE1 ori: replication origin of *E*. *coli* ColE1 plasmid.

For plasmid propagation, the *E*. *coli* DH5α strain was used as a host, and strains were grown with constant agitation at 37°C in Lysogeny Broth (LB) medium supplemented with 100 μg/ml ampicillin and 40 μl of a 2% solution of X-gal (5-bromo-4chloro-3-indolyl-β-D-galactopyranoside) for plasmid and recombinant clone selection, respectively [30]. For heterologous expression of the *X*. *dendrorhous MIG1* gene in *E*. *coli*, the BL21 (DE3) PLysS strain was used and grown in LB medium supplemented with 50 μg/ml kanamycin.

*X*. *dendrorhous* strains were grown at 22°C with constant agitation in YM medium (0.3% yeast extract, 0.3% malt extract and 0.5% peptone) supplemented, when indicated, with 1% glucose (YM-1% glucose) or in Vogel minimal medium (MMv) supplemented either with 2% glucose or maltose [26]. Transformant selection was performed on YM-1% glucose-1.5% agar plates containing 15 μg/ml hygromycin B.

*S*. *cerevisiae* strains were cultured at 22°C or 30°C in YEP medium (1% yeast extract, 2% peptone, 2% glucose) or SD minimal medium (0.67% Yeast Nitrogen Base (Difco, Becton, Dickinson and Company, Franklin Lakes, NJ, USA) and 2% glucose) supplemented with uracil (0.02 g/l), histidine (0.02 g/l), methionine (0.02 g/l) or leucine (0.1 g/l), according to individual strain auxotrophies. For heterologous complementation assays, the ability of strains to grow with a sole carbon source in the presence of the non-hydrolyzable glucose analog 2-deoxy-D-glucose (2DG) was evaluated on SD agar plates supplemented with amino acids according to mutant strain requirements, with the exception of leucine, as the expression vector complements this strain auxotrophy. Additionally, 2% glucose, raffinose or sucrose was used as the sole carbon source in addition to 0.02% 2DG. Plates were incubated for 5 days at 22°C given that this is the optimal growth temperature of *X*. *dendrorhous* and the gene under evaluation is native to this yeast.

Culture curves were constructed in triplicate at a minimum, and growth was registered by measuring the optical density of the cultures at 600 nm (OD_600_) using a V-630 UV-Vis spectrophotometer from JASCO (JASCO, Easton, MD, USA). Biomass was quantified gravimetrically by measuring the dry weight of 1 ml of yeast culture in triplicate. The glucose remaining in the culture medium was determined using the DNS (dinitrosalicylic acid) method [37] by mixing the sample with the DNS reagent (1% DNS, 1.6% NaOH and 15% Na and K tartrate) at a ratio of 1:1 and incubating at 100°C for 10 min. Reactions were halted by incubating the mixture on ice, and the absorbance at 540 nm was measured. Glucose concentration was determined from a standard curve (absorbance at 540 nm vs. glucose concentration) that was previously constructed.

### Invertase activity

Extracellular invertase activity measurements were performed as previously described [38]. *X*. *dendrorhous* extracellular invertase activity was measured in culture supernatant samples that were mixed 1:1 with 50 mM sodium acetate buffer at pH 5.4 with or without (as control) 2% sucrose, incubated at 45°C for 30 min and then maintained at -20°C. To evaluate invertase activity in the heterologous complementation assays in *S*. *cerevisiae*, the yeast strains were cultured at 22°C in SD minimal medium supplemented with galactose for two days, and then cells were washed and divided into two fractions consisting of SD medium containing 2% galactose supplementation with or without 2% glucose. Both fractions were incubated at 22°C for 5 h, and cells were harvested, washed with water and incubated for 5 min at 30°C in 50 mM sodium acetate buffer at pH 5.4 with or without 2% sucrose.

Units of invertase activity were defined as g of glucose released/g of dry yeast weight, and the glucose released from sucrose during the reactions was determined using the DNS method [37].

### Standard molecular biology procedures, nucleic acid extraction, PCR reactions, synthesis of cDNA and qPCR

Standard procedures (PCR reactions, restriction enzyme digestions, ligation reactions and *E*. *coli* transformations) were performed according to standard protocols [30]. The primers used in this work are listed in S1 Table. The general PCR protocol was: initial denaturation at 95°C for 3 min; 35 cycles of denaturation at 94°C for 30 s, annealing at 55°C for 30 s, and synthesis at 72°C for 3 min; and a final extension step at 72°C for 10 min.

Total DNA was extracted from the yeasts *X*. *dendrorhous* and *S*. *cerevisiae* as previously described [39], and total RNA was obtained from *X*. *dendrorhous* using the yeast RiboPure RNA Purification Kit (Ambion, Thermo Fisher Scientific Inc., Waltham, MA, USA) according to the manufacturer's recommendations. The AxyPrep Plasmid Miniprep Kit (Axygen, Corning Incorporated, Corning, NY, USA) were used for *E*. *coli* plasmid DNA extraction and purification. The integrity and concentration of genomic DNA, plasmids, RNA and PCR products were analyzed by performing 0.7–1% agarose gel electrophoresis in 1X TAE buffer (40 mM Tris-acetate, 1 mM EDTA mM, pH 8.0). Gels were stained with 0.5 μg/ml ethidium bromide and visualized under UV light. When necessary, DNA was purified from gels using the Ultra Clean 15 DNA Purification Kit (MO BIO Laboratories Inc., Carlsbad, CA, USA) following the manufacturer's protocol.

Nucleotide sequences were obtained with an ABI 3100 Avant genetic analyzer using the BigDye Terminator v3.1 Kit (Applied Biosystems, Thermo Fisher Scientific Inc., Waltham, MA, USA) or via services from Macrogen Inc. (Seoul, South Korea). DNA sequences were analyzed with CLUSTAL W 1.8, Geneious 8.1.3 and programs available at the NCBI web site. The prediction of regulatory sequences in gene promoters was performed using the PROMO 3.0 application available online (http://alggen.lsi.upc.es/cgi-bin/promo_v3/promo/promoinit.cgi?dirDB=TF_8.3), which employs matrices of consensus sequences of regulatory binding sites deposited in the transcription factor database TRANSFAC [40,41]. The TRANSFAC code used for the Mig1 transcription factor was T00509. In addition, a manual search was performed using the CreA consensus binding sequence described previously for *Aspergillus nidulans* [42], which was defined as (G/C)(C/T)GG(A/G)G.

Synthesis of cDNA was carried out in a final volume of 20 μl using 5 μg of total RNA, 25 μM oligo-dT18, 1.25 μM dNTPs and 200 U of 0.5 μM reverse transcriptase M-MLV (Invitrogen, Thermo Fisher Scientific Inc., Waltham, MA, USA), according to the enzyme manufacturer’s protocol. Determination of the relative transcript levels was performed by mixing 1 μl of the reverse transcription reaction, 0.25 μM of each primer and 10 μl of the SensiMix SYBR Green I kit (Bioline, London, UK) in a final volume of 20 μl, followed by analysis on an Mx3000P quantitative PCR system (Stratagene, Agilent Technologies Inc., Santa Clara, CA, USA). The obtained Ct values were normalized to the values for ß-actin, encoded by *ACT* [GenBank: X89898.1] [43] and were later expressed as a function of the control conditions using the algorithm 2^-ΔΔCt^ [44].

Transcriptomes of the wild-type and *Xdmig1*^*-/-*^ strains were obtained from RNA samples taken from cultures during the early exponential phase of growth (36 h of culture) using MMv medium supplemented with 2% glucose. When samples were taken, 1% glucose was present in the culture media. RNA-seq was performed by Macrogen Inc. (Seoul, South Korea) using an Illumina HiSeq2000 System with a 100-bp paired-end library as described previously [45]. The raw transcriptomic data were analyzed with CLC Genomics Workbench 5, and the identification of differentially expressed genes (DEGs) between the wild-type and *Xdmig1*^*-/-*^ mutant strains was performed using the DESeq2, EdgeR and EBSeq statistical analysis packages for R and Rstudio, which are available online (https://cran.r-project.org and www.rstudio.com, respectively).

### Electrophoretic Mobility Shift Assays (EMSAs)

EMSAs were performed with the purified Mig1 protein from *X*. *dendrorhous*, which was expressed in *E*. *coli* (DE3) PLysS using the plasmid pET-TEV, which adds a His-tag to the recombinant protein. Gene expression was induced with 100 mM IPTG using exponential growth cultures in SOC media containing corresponding antibiotics. After induction, cells were collected, washed and suspended in buffer A (20 mM Tris, 50 mM NaCl, 20 mM Imidazole, 0.1% Triton x100; pH 8.0). Cells were broken using a Cole Parmer 4710 ultrasonic homogenizer and then centrifuged at 13.150 x g for 10 min at 4°C. Once the presence of the recombinant protein was confirmed in relation to the control by SDS-PAGE analysis, the protein band was excised from the gel and analyzed by MALDI-TOF (CEPDEQ Universidad de Chile, Santiago, Chile) to confirm the identity of the protein. The protein was purified using agarose Ni-NTA resin (Qiagen, Hilden, Germany), and the His-tag was then removed by TEV protease (Sigma-Aldrich, St Louis, MO, USA) treatment. Protein quantification was carried out using the Bradford method.

EMSAs were performed using the promoter region of *X*. *dendrorhous* genes with predicted “Mig1 boxes”. Oligonucleotides containing the corresponding “Mig1 boxes” were labeled with biotin at their 5’ end, including the genes *grg2* [2 DNA fragments containing box A or box B, primer pairs: grg2.1-348.Fw + grg2.1-348.Rv (54 bp) and grg2.1-220.Fw + grg2.1-220.Rv (53 bp), respectively], *crtI* [1 DNA fragment, primer pair PcrtI.1000.Fw + crtI.Mig1.Rv (249 bp)], *crtYB* [1 DNA fragment, primer pair PcrtYB.1000.Fw + crtYB.Mig1.Rv (254 bp)] and *crtS* [3 DNA fragments containing box A, boxes B-C or boxes C-D, primer pairs: crtS1000.Fw + crtS.Mig1-854.Rv (183 bp), PS1Fw + PS1Rv (204 bp) and PS2Fw + PS2Rv (209 bp), respectively].

dsDNA samples were obtained after oligonucleotide hybridization in buffer A (10 mM Tris-HCl, 1 mM EDTA, 50 mM NaCl; pH 8.0) under the following conditions: denaturation at 95°C for 5 min and 70 temperature-decreasing cycles from 95 to 25°C lasting 1 min. DNA-protein binding reactions contained 20 fmol of labeled dsDNA, union buffer 1X (20 mM HEPES, 1 mM DTT, 0.1 mM EDTA, 50 mM KCl, 5% glycerol, 200 μg/ml BSA; pH 7.9), 2.5% glycerol, 50 ng/μl Poly (dI•dC), 0.05% NP-40 and 10 μg of purified Mig1 protein in a final volume of 20 μl. As a control, the purified protein was incubated with non-labelled DNA before its incubation with the labelled DNA. The mixtures were incubated at room temperature for 20 min, and then 5 μl of loading buffer was added for gel loading. Electrophoresis was performed for 1 h at a constant 100 V in cold TBE (45 mM Tris-borate, 0.45 mM boric acid, 0.1 mM EDTA) 0.5X buffer in a 6% polyacrylamide-TBE 0.5X gel, which was previously run at 100 V for 1 h at 4°C. The resolved samples were transferred to a nylon membrane that was previously incubated in TBE 0.5X at 4°C for 10 min with Trans-Blot Semi-Dry (BIORAD, Hercules, CA, USA) under the following conditions: 5 V for 5 min, 10 V for 10 min, 15 V for 10 min and 20 V for 5 min. DNA was immobilized by UV (312 nm) exposition for 10 min, and the labeled DNA was visualized with the LightShift Chemiluminescent EMSA Kit (Thermo Scientific, Thermo Fisher Scientific Inc., Waltham, MA USA) following the supplier’s instructions.

### Plasmid construction and yeast transformation

All plasmids used and constructed in this study are listed in Table 1. The *X*. *dendrorhous MIG1* gene from the wild-type strain UCD 67–385 was identified by BLAST analyses of local genomic and transcriptomic databases [45]. Based on the identified gene sequences, primers were designed for plasmid construction and yeast transformation.

The complete *X*. *dendrorhous MIG1* cDNA (2,592 bp) was obtained by overlap extension PCR (OE-PCR) of two cDNA fragments approximately 1,100 and 1,600 bp in size, which were obtained by RT-PCR from total *X*. *dendrorhous* RNA using the primer pairs Mig1cDNA+Mig1cDNARV2 and Mig1_Fw1480+Mig1cDNA_Stop. The OE-PCR product was inserted at the *Eco*RV site of the pBluescript plasmid, resulting in the plasmid pXdcMIG1.

For *S*. *cerevisiae* heterologous complementation assays, the *X*. *dendrorhous MIG1* cDNA was PCR-amplified from the plasmid pXdcMIG1 and inserted at the *Xba*I site of plasmid YEp-NP, resulting in the plasmid YEp-NP-MIG1. The correct orientation of the DNA fragment in the expression plasmid was confirmed by PCR. *S*. *cerevisiae* electrocompetent cells were transformed with a BioRad Gene Pulser Xcell with PC and CE modules using 1.5 kV, 25 μF and 200 Ω.

To study *MIG1* gene functionality in *X*. *dendrorhous*, the *Xdmig1*^*-/-*^ strain was obtained via the DNA assembler technique [46,47]) followed by the Double Recombination Method (DRM, [48]). For this, an “up” fragment (307 bp) and a “down” fragment (393 bp) at the *MIG1* locus were PCR-amplified using primers Mig1up2.F+10mig.up2-50TEF.R and 50.IIMig1.dw-10gpd.T.F+IIMig1.dw.R (S1 Table). Both amplified regions were spaced 640 bp apart in the yeast genome; the 640-bp span included 96 bp of the gene promoter and the first three exons of the gene that encodes the zinc finger motif of the *X*. *dendrorhous* Mig1 protein. Additionally, a module that confers resistance to hygromycin B in *X*. *dendrorhous* was amplified from plasmid PMN-Hyg [33]. The primers used to amplify the three DNA fragments, the “up” fragment, the resistance module and the “down” fragment, were designed with 5’ complementary ends to generate PCR products with overlapping sequences of 100 bp between the downstream region of the “up” fragment and the upstream region of the resistance module and between the downstream region of the resistance module and the upstream region of the “down” fragment. In this way, the three DNA fragments would assemble *in vivo* and integrate at the *MIG1* locus by homologous recombination after yeast transformation [47] to give rise to a *mig1*^-^ mutant.

*X*. *dendrorhous* transformation was performed by electroporation using electrocompetent cells obtained from exponential phase growth cultures (OD_600nm_ = 1.2) developed in YM-1% glucose medium [33]. Transformations were performed using 1 to 5 μg of linear donor DNA under the following conditions: 125 mF, 600 Ω, 0.45 kV. Transformants were selected on YM-1% glucose- 1.5% agar plates supplemented with 15 μg/ml hygromycin B. As strain UCD 67–385 is diploid [49], heterozygous transformants mutants were obtained. To obtain a homozygous mutant, the double recombinant method [48] was applied to a heterozygous *mig1*^-^ mutant. Transformants were confirmed to be *X*. *dendrorhous* by sequence analysis of the ITS1, 5.8 rRNA gene and ITS2 DNA sequences [50].

### Carotenoid extraction and analysis

Carotenoids were extracted from cellular pellets using the acetone extraction method [51] and quantified spectrophotometrically at 474 nm. An absorption coefficient of A_1%_ = 2,100 was used, and data were normalized to the yeast dry weight. To analyze the carotenoid composition, pigment extracts were separated by RP-HPLC using a reverse phase 125–4 LiChrospher RP-18 column (Merck Millipore, Billerica, MA, USA) with acetonitrile:methanol:isopropanol (85:10:5 v/v) as the mobile phase with a 1 ml/min flux rate under isocratic conditions [28]. Elution absorption spectra were acquired using a Shimadzu SPD-M10A diode array detector (Shimadzu, Kyoto, Japan), and carotenoids were identified by comparing their retention times and absorption spectra with known standards [52].

## Results and Discussion

### Catabolic repression in *X*. *dendrorhous*

**Fig 2:**
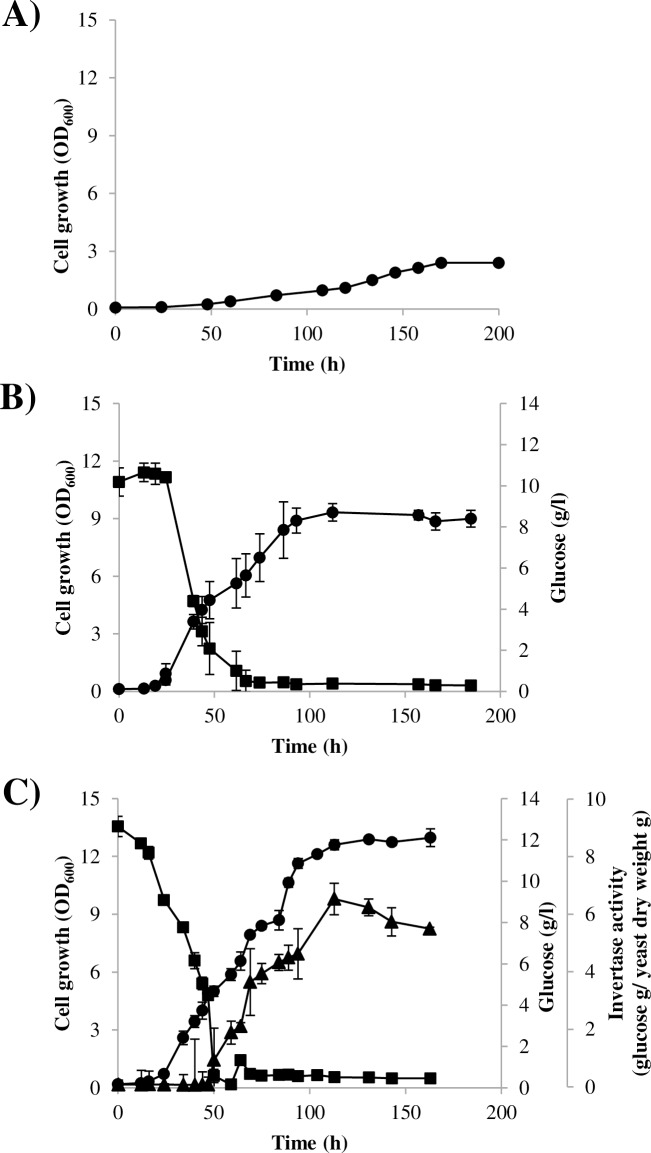
Growth curve and extracellular invertase activity of the *X*. *dendrorhous* wild-type strain cultured with sucrose and/or glucose as a carbon source. Growth curves of the UCD 67–385 wild-type strain cultured at 22°C with constant agitation in MMv medium supplemented with A) sucrose (10 g/l), B) glucose (10 g/l) and C) glucose (10 g/l) and sucrose (10 g/l) are represented. Yeast growth, residual glucose in the culture medium and invertase activity are represented in circles, squares and triangles, respectively. Values correspond to the average of three independent cultures, and error bars correspond to the standard deviation.

As previously mentioned, the expression of yeast genes involved in the use of alternative carbon sources other than glucose is usually inhibited by catabolic repression. Thus, to assess whether this process is an operative mechanism in *X*. *dendrorhous*, the wild-type yeast strain was cultured in MMv supplemented either with glucose or sucrose or with both sugars (Fig 2). Extracellular invertase activity was assayed during growth as this activity was not previously detected in a glucose-based medium, suggesting its catabolic repression [29], which occurs in other organisms [13]. When the yeast was cultured using glucose as a carbon source, the generation time was 8.7 ± 0.1 h, and approximately 10 units at OD_600nm_ was reached in the stationary phase of growth. In contrast, a generation time of 36.6 ± 0.02 h and a maximum OD_600nm_ of approximately 2.5 units were achieved when the yeast was cultured on sucrose as the sole carbon source. Interestingly, using medium containing a mixture of both sugars, the growth curve exhibited diauxic behavior in which the maximum OD_600nm_ reached during the first growth phase, when glucose was consumed, coincided with the maximum OD_600nm_ reached in cultures supplemented only with glucose. A diauxic growth response reflects the sequential use of two available sugars: glucose is used first, and the use of the second carbon source is limited until glucose is exhausted. This is generally achieved through glucose-dependent catabolic repression of the genes required for the transport and/or metabolization of the alternative sugar [53]. Accordingly, extracellular invertase activity was detected only after the culture was depleted of glucose; at that point, invertase activity increased during growth, suggesting that invertase activity is repressed in presence of glucose. These results clearly demonstrate that *X*. *dendrorhous* contains an operative catabolic repression mechanism.

### Identification and analysis of the *X*. *dendrorhous MIG1* gene

**Fig 3:**
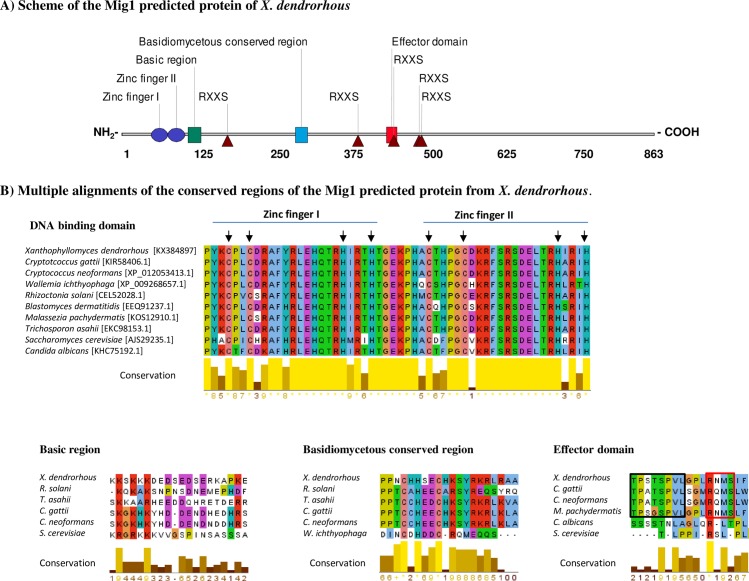
Sequence analyses of the deduced Mig1 protein from *X*. *dendrorhous*. A) Illustrative scheme of the deduced Mig1 protein from *X*. *dendrorhous*; conserved features that are important for protein function are highlighted. The RXXS feature represents potential Snf1 phosphorylation sites. B) Multiple alignments of the conserved regions in the Mig1 protein from *X*. *dendrorhous* and from other yeasts and filamentous fungi. Upper: DNA binding domain; the cysteine and histidine residues in the zinc finger motif are indicated with arrows. Lower left: alignment of the basic conserved region. Lower middle: alignment of the basidiomycete conserved region. Lower right: alignment of the effector domain; the amino-terminal region characteristic of Mig1 in basidiomycetes and the potential Snf1 phosphorylation site are enclosed in black and red squares, respectively. Residues were colored with the Clustal default color key, which assigns a color according to the residue physicochemical properties and similitude profile in the alignment. The degree of conservation of each residue is represented by a scale from 0 to 10. The GenBank or NCBI reference sequence accession numbers are included in square brackets in the first alignment.

Given that the Mig1 protein fulfils a major role in catabolic repression and that carotenogenesis in *X*. *dendrorhous* may be regulated by this mechanism, the hypothetical *MIG1* gene from *X*. *dendrorhous* was searched in genomic and transcriptomic databases for the wild-type yeast [45]. BLAST analyses employing previously described Mig1 proteins from other organisms as queries resulted in the identification of the potential *X*. *dendrorhous MIG1* gene [GenBank: KX384897]. The sequence of the deduced Mig1 protein shared 53% identity and 33% coverage with the CreA DNA-binding protein (the Mig1 homolog) from *Cryptococcus gattii*. The *MIG1* gene structure was deduced by comparing its genomic and cDNA sequences; it contained 4 exons that were 94, 95, 159 and 2,244 bp in size, resulting in an ORF of 2,592 bp that encoded an 863-residue polypeptide with a predicted molecular weight of 92.24 kDa. Multiple sequence alignment using Mig1 proteins from related species to identify conserved features that are relevant to the function of this transcription factor were identified in the deduced *X*. *dendrorhous* protein (Fig 3). Among them, the type C_2_H_2_ zinc finger motif necessary for DNA binding was identified between residues 46 and 100. Eight RxxS motifs that are potential phosphorylation targets of the Snf1 kinase, which regulates Mig1 cellular localization, and a conserved region that corresponds to the effector domain originally described in the *S*. *cerevisiae* Mig1 protein [54] were identified. In addition, a conserved region rich in basic residues and other regions conserved in Mig1 proteins from basidiomycetes were identified (Fig 3). The presence of these conserved features in the deduced protein supports the notion that the identified *MIG1* gene encodes a functional Mig1 protein.

In previous studies, potential Mig1 boxes were identified in the promoter regions of the *X*. *dendrorhous* carotenogenic genes *crtYB* (CCCCAAAATCA), *crtI* (CCCACAGATA) and *crtS* (CTGGAG, ACGAGGTGGGG, CTGGGCTGGGG and CTGGAG; boxes A, B, C and D, respectively) [26] and in the glucose-repressed gene *grg2* (GCGGAC and GCGGAG; boxes A and B, respectively) of unknown function, which was used as a glucose-repressed gene control [28]. To confirm whether the *X*. *dendrorhous MIG1* gene product binds to DNA containing Mig1 boxes, EMSAs were performed using biotin-labeled DNA fragments of the promoter regions of the *crtI*, *crtYB*, *crtS* and *grg2* genes containing the potential Mig1 boxes.

The EMSAs were initially performed with purified protein and biotin-labeled DNA fragments approximately 60 bp in length, including the predicted Mig1 boxes of the promoter regions of the *grg2*, *crtI*, *crtYB* and *crtS* genes. In this way, an electrophoretic mobility shift was only observed when using a DNA fragment containing one (GCGGAG, box A) of the two predicted Mig1 boxes in the promoter region of the *grg2* gene. It is likely that any other 60-bp DNA fragments containing a Mig1 box were not sufficient to allow protein binding and subsequent mobility shift. Supporting this hypothesis, previous reports have indicated that the DNA sequences adjacent to a Mig1 box are highly important for the functionality of this element; specifically, upstream regions that contain a greater AT content than a GC content permit protein-DNA interactions [55]. Thus, the *MIG1* gene product failed to bind to the assessed regulatory regions of the carotenogenic genes of *X*. *dendrorhous*, possibly due to a lack of additional regions needed for protein binding. With these considerations, additional EMSA analyses were performed using larger DNA fragments of the promoter regions of the carotenogenic genes *crtI* (1 DNA fragment), *crtYB* (1 DNA fragment) and *crtS* (3 DNA fragments containing box A, boxes B-C or box C-D), including the deduced Mig1 boxes and approximately 140 to 160 bp of the upstream region of one of these elements, resulting in a DNA fragment approximately 200 bp in length. Although only the upstream region of the Mig1 box of the *crtYB* gene has a greater AT content, all DNA fragments demonstrated electrophoretic mobility shifts when they were incubated with purified Mig1 protein but not when the protein was previously incubated with non-labelled DNA (S1 Fig), indicating that the Mig1 protein binds to these DNAs.

### Mutation of the *X*. *dendrorhous MIG1* gene

To study the functionality of the identified gene, a *X*. *dendrorhous Xdmig1*^*-/-*^ mutant was obtained to evaluate the effects of mutation on yeast growth, carotenoid production, gene expression, and extracellular invertase activity.

#### Effects of *X*. *dendrorhous MIG1* gene mutation on growth

Growth curves for the wild-type and *Xdmig1*^*-/-*^ mutant strains were obtained using YM-1% glucose medium and MMv medium supplemented with 2% glucose (S2 Fig). In both media, the *Xdmig1*^*-/-*^ mutant strain reached a significantly higher OD_600_ during the stationary phase of growth compared to the wild-type strain (maximum OD_600_ reached in YM-1% glucose and MMv + 2% glucose media: approximately 10–11 and 8 for the wild-type strain versus approximately 14–17 and 15–16 for the *Xdmig1*^*-/-*^ mutant in both media, respectively). The generation times calculated for both strains were also significantly different (Student’s t test, p ≤ 0.01), being 5.2 ± 0.2 and 7.9 ± 0.1 h in YM-1% glucose and MMv supplemented with 2% glucose media, respectively, for the wild-type strain in contrast to the *Xdmig1*^*-/-*^ mutant strain, which were 4.7 ± 0.1 (in YM-1% glucose) and 7.4 ± 0.1 h (in MMv with 2% glucose). The differences detected in these growth parameters demonstrate that the *mig1*^-^ mutation alters the general physiology of the yeast. This is consistent with previous observations in *S*. *cerevisiae* in which *MIG1* disruption caused an increase in the growth rate of the yeast when cultured with glucose, which could be explained by the derepression of genes involved in glucose uptake, such as certain *HXT* genes resulting in an augmented influx of glucose [56]. Another possibility may be the derepression of genes involved in respiratory functions favoring biomass production; this explanation explains the increased growth rate observed in a *S*. *cerevisiae* strain overexpressing the *HAP4* gene, which encodes an activator of respiratory genes [56,57].

#### Effects of *X*. *dendrorhous MIG1* gene mutation on carotenoid production

As mentioned above, experimental evidence strongly suggests that carotenoid production in *X*. *dendrorhous* may be regulated by catabolic repression in which the *MIG1* gene product plays a central role. Thus, to evaluate whether carotenoid production is affected in the *Xdmig1*^*-/-*^ mutant strain, samples were taken from wild-type and *Xdmig1*^*-/-*^ strain cultures grown in YM-1% glucose medium at 5 different time points representative of different growth phases (Table 2). Carotenoid content was higher in the *Xdmig1*^*-/-*^ mutant strain during almost all phases evaluated, and the total carotenoid content in the final phase of growth evaluated was approximately 20% higher in the *Xdmig1*^*-/-*^ strain compared to the wild-type. Carotenoid composition was similar in both strains with the exception of the early exponential phase, during which the fraction of astaxanthin was higher in the *Xdmig1*^*-/-*^ mutant. The higher carotenoid production in the *X*. *dendrorhous Xdmig1*^*-/-*^ mutant strain strongly suggests a role for Mig1 in the regulation of carotenogenesis in this yeast.

**Table 2:** Carotenoid content and astaxanthin proportion in the wild-type and *Xdmig1*^*-/-*^ strains during different growth phases.

	Carotenoid content (μg carotenoids/g yeast dry weight) and astaxanthin proportion (%).				
Strain:	S1	S2	S3	S4	S5
**Wild-type**	29 ± 3	64 ± 4	195 ± 8	271 ± 12	338 ± 6
	(41)	(44)	(47)	(60)	(56)
***Xdmig1***^***-/-***^	51 ± 5**	100 ± 2*	220 ± 9	354 ± 12**	412 ± 17**
	(61)	(56)	(55)	(57)	(61)

S1 to S5 represent samples from 5 different growth phases: S1: early exponential (24 h), S2: half exponential (36 h), S3: late exponential (48 h), S4: early stationary (65 h) and S5: late stationary (105 h). The percentage of astaxanthin in each sample is indicated in parenthesis. Student’s t test was performed to assess the results from the wild-type and *Xdmig1*^*-/-*^ strains; significant differences are indicated for values obtained from strain *Xdmig1*^*-/-*^ (*p ≤ 0.01; **p ≤ 0.05).

**Fig 4:**
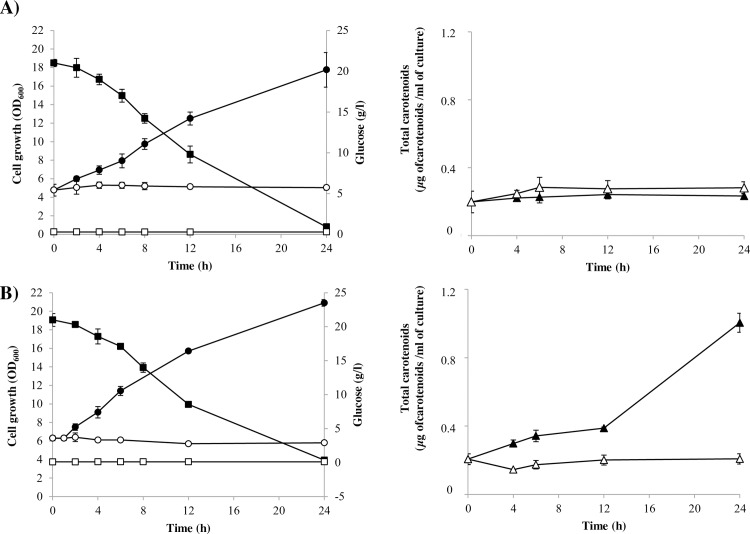
Effects of glucose on growth and carotenoid production in the wild-type and *Xdmig1*^*-/-*^ strains. A) data from the wild-type; B) data from strain *Xdmig1*^*-/-*^. The glucose-treated (final concentration of 20 g/l) and control conditions results are represented in black or white symbols, respectively. On the left, residual glucose (squares) and yeast growth (circles); on the right, total carotenoid content (μg carotenoids/ml) are represented. Values correspond to the average of three independent cultures, and error bars represent the standard deviation.

To evaluate if the observed differences in carotenoid content between the wild-type and the *Xdmig1*^*-/-*^ strain could be attributable to an alleviation of glucose-mediated repression of carotenogenesis in the latter, both strains were cultured in YM medium without glucose supplementation. When the cultures reached the stationary phase of growth, each one was divided between two flasks: one of them was supplemented with glucose (at a final concentration of 20 g/l) and the other one was left as a control without glucose supplementation [28]. Then, the new cultures were incubated at 22°C and growth, residual glucose and carotenoid content profiles were analyzed (Fig 4). Total RNA was also extracted from samples obtained from the *Xdmig1*^*-/-*^ strain to evaluate the transcript levels of selected genes by RT-qPCR.

**Fig 5:**
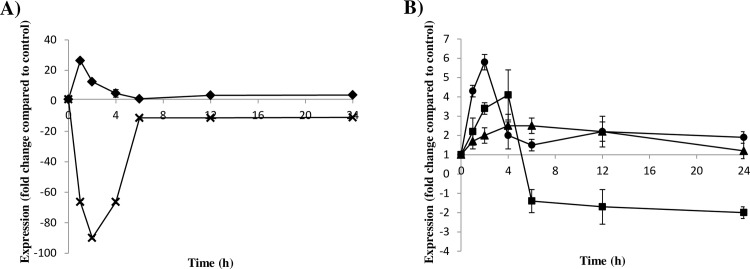
Relative transcript levels of the carotenogenic genes *crtYB*, *crtI* and *crtS* in the *Xdmig1*^*-/-*^ mutant strain after glucose treatment. The expression kinetics of the mRNA levels of A) the *grg2* (crosses) and *PDC* (rhomboids) control genes and B) the carotenogenic genes *crtYB* (circles), *crtI* (squares) and *crtS* (triangles) in strain *Xdmig1*^*-/-*^ after glucose addition (final concentration of 20 g/l) relative to the control fraction (without glucose) are indicated. Data represent the mean of three independent experiments, and the error bars correspond to the standard deviation.

As expected, no glucose was detected in the medium from the control cultures of both strains, while glucose was detected in both glucose-treated cultures, and its concentration decreased over time (Fig 4A). Unlike the control cultures, growth was observed in both strain cultures after the addition of glucose (Fig 4B). At all analyzed time points, the total carotenoid content did not change in either control culture, but, upon glucose addition, an increase in the total carotenoid content was observed only in *Xdmig1*^*-/-*^ strain cultures (Fig 4C). An evident difference in the total carotenoid content between the *Xdmig1*^*-/-*^ and the wild-type strain is observed 12 h after glucose addition (time point at which glucose was depleted from the culture medium); however, this is not surprising as there must be a delay between carotenoid production, carotenogenic genes transcription and protein synthesis. Then, the carotenoid production observed in *Xdmig1*^*-/-*^ strain cultures after 12 h post glucose addition, should be the result of higher transcript level of carotenogenic genes earlier in the culture when glucose concentration is still high in the culture medium (see below, Fig 5). In contrast to the *Xdmig1*^-^ strain, in the wild-type strain no carotenoid production was observed after glucose addition during the 24 h studied, suggesting that the derepression of the carotenogenic genes in the *Xdmig1*^-^ mutant strain, takes place earlier than in the wild-type.

These results indicate that glucose is consumed and its addition restarts growth in both strain cultures; however, glucose-mediated repression of the synthesis of carotenoids is only observed in cultures from the wild-type strain.

#### Effects of *X*. *dendrorhous MIG1* gene mutation on gene expression

In addition to the inhibitory effects of glucose on carotenoid production, it was previously reported that glucose exerts a repressive effect on the transcript levels of carotenogenic genes in the wild-type strain [28]. Given this, the effects of glucose on *crtI*, *crtYB* and *crtS* transcript levels were evaluated in the *Xdmig1*^*-/-*^ strain by RT-qPCR using total RNA extracted from glucose-treated and control cultures (Fig 5). The obtained results were compared to those previously reported for the wild-type strain employing the same experimental approach [28]. The *grg2* and *PDC* genes were included in the analyses as controls for glucose-mediated repression and induction, respectively. In the case of the *grg2* gene, up to an approximately 90-fold decrease in mRNA levels was observed in the glucose-treated *Xdmig1*^*-/-*^ strain sample; this value is lower than that one reported for the wild-type strain (130-fold decrease) [28]. It is possible that the residual repressive effects of glucose on the *grg2* gene in the *Xdmig1*^*-/-*^ mutant strain may be attributable to redundant regulatory mechanisms modulating the expression of this gene of unknown function. As expected, the mRNA levels of the *PDC* gene increased after glucose addition, and no differences were observed between *Xdmig1*^*-/-*^ and the previously reported data from the wild-type strain. In contrast, the transcript levels of the carotenogenic genes *crtI*, *crtYB* and *crtS* in the *Xdmig1*^*-/-*^ mutant strain increased after glucose addition (Fig 5), unlike the wild-type strain, in which the transcript levels of these genes decreased after glucose treatment [28]. Notably, this is consistent with the presence of Mig1 boxes in the promoter regions of these three genes, which bind the purified recombinant Mig1 protein from *X*. *dendrorhous in vitro*. In this yeast, two mRNA versions (mature and alternative) of the *crtI* and *crtYB* genes have been described [58], but given that only the mature versions encode functional products in both cases, only the mature version of each transcript was considered in this study. In the case of mature *crtI* gene transcript levels, an increase of approximately 4-fold was observed in the *Xdmig1*^*-/-*^ mutant strain after glucose addition, compared to a 6-fold decrease reported for the wild-type strain. This indicates that the glucose-dependent repression of this gene was mitigated in the *Xdmig1*^*-/-*^ mutant strain. Similarly, the mature *crtYB* and the *crtS* transcript levels in the *Xdmig1*^*-/-*^ mutant strain increased up to approximately 5-fold and 2-fold, respectively, after glucose treatment. Conversely, the abundance of both transcripts decreased up to approximately 6-fold and 4-fold, respectively, in the wild-type strain after glucose treatment [28]. Together, these results support the notion that Mig1 regulates carotenogenesis by affecting the transcripts levels of carotenogenic genes, likely as part of a larger catabolic repression mechanism, which can explain the increased carotenoid production observed in the *Xdmig1*^*-/-*^ mutant in cultures using glucose as a carbon source.

To further corroborate the results described above and to identify putative additional Mig1-regulated genes, the transcriptomes of the wild-type and *Xdmig1*^*-/-*^ strains cultured with glucose as the sole carbon source were analyzed. As a first approximation, the promoter regions of potential glucose-regulated genes were searched to identify putative Mig1 boxes using the PROMO 3.0 application or the previously defined consensus sequence (G/C)(C/T)GG(A/G)G [42]. These genes were identified in a local genomic database using known catabolic repression target genes primarily described in *S*. *cerevisiae* as references [13,59]. Additionally, the carotenogenic *crtI*, *crtYB* and *crtS* genes, as well as the control genes *grg2*, *PDC* (both under glucose regulation) and *ACT* (constitutive gene), were included in the analysis. Moreover, the expression of the different transcripts was estimated by calculating their RPKM (Reads Per Kilobase per Million mapped reads) values [60] (Table 3). As previously reported, at least one Mig1 box-type sequence was identified in the promoter regions of the three carotenogenic genes analyzed, and four potential Mig1 boxes were identified in the *crtS* gene promoter. This result is in accordance with the lower RPKM values obtained for the carotenogenic gene transcripts in the wild-type strain transcriptome compared to the values obtained in the *Xdmig1*^*-/-*^ transcriptome. These results are also in agreement with those obtained by RT-qPCR, supporting the notion that Mig1 may regulate the expression of carotenogenic genes at the transcriptional level. On the other hand, among the studied genes with potential Mig1 boxes in their promoters, only 6 demonstrated a higher RPKM value in the mutant strain compared to the wild-type (Table 3). This result suggests that in *X*. *dendrorhous*, a redundant glucose-dependent regulation mechanism may exist, as observed in *S*. *cerevisiae*, in which the Mig2 regulator has a similar function to Mig1 but is activated through a different signaling cascade [13,61]. It is also possible that the expression of some of the studied genes might require specific activation in addition to the release of Mig1 repression; thus, the *mig1*^*-*^ mutation is not sufficient for constitutive transcription. In the case of the *grg2* control gene, only a small difference between the RPKM values in the wild-type and mutant strain was observed, which is consistent with the RT-qPCR results (Fig 5), demonstrating that the *mig1*^*-*^ mutation did not completely alleviate *grg2* glucose-mediated repression. Finally, as expected, both the *PDC* and *ACT* control genes did not exhibit differences in their RPKM values between both strains when cultured with glucose; these genes would not be Mig1 targets.

**Table 3:** Bioinformatic analysis of potential glucose-regulated genes in *X*. *dendrorhous*.

				RPKM		
Gene/product [GenBank N°]	Biological process	Mig1 box sequence	Upstream position*	Xd*mig1*^-/-^ (mut)	wild-type(wt)	Ratio (mut/wt)
*crtS*/astaxanthin synthase [DQ201828.1]	Carotenoid synthesis	CTGGAG	-469	**116.6**	**59.3**	**2.0**
		CTGGGCTGGGG			
		ACGAGGTGGGG	-749			
		CTGGAG	-847			
*crtYB*/phytoene β-carotene synthase [DQ016503]	Carotenoid synthesis	CCCCAAAATCA	-774	**57.1**	**27.2**	**2.1**
*crtI*/phytoene desaturase [DQ028748.1]	Carotenoid synthesis	CCCACAGATA	-777	**193.3**	**128.0**	**1.5**
*IDH*/isocitrate dehydrogenase [KX384884]	TCA cycle	TCCATCTGGGG	-312	120.3	146.1	0.8
*CIT1*/citrate synthase [KX384887]	TCA cycle	TTATGTTGGGG	-1,555	668.8	944.0	0.7
		CCCCAACCACT	-1,690			
*COX6*/cytochrome C oxidase [KX384886]	Mitochondrial electron transport	CCCCATATTTC	-483	796.2	831.5	1.0
*CYC1*/cytochrome C1 [KX384888]	Mitochondrial electron transport	CCCCAATCCAA	-327	2,733.8	2,859.5	1.0
*GUT1*/glycerol kinase [KX384889]	Glycerol metabolism	GCTCGGTGGGG	-126	45.7	57.8	0.8
		CCCAGGGTTG	-832			
		CCCCAGTCCTG	-1,490			
*GUT2*/glycerol 3 phosphate dehydrogenase [KX384890]	Glycerol metabolism	CCCCAAAGAGC	-2,295	58.7	132.6	0.4
*MAL5*/glucan 1,4 α-glucosidase [KX384891]	Carbohydrate catabolism	CCCCATATGAA	-610	46.8	47.4	1.0
		TGGAAGTGGGG	-1,154			
*AMY1*/α-amilase [KX384895]	Carbohydrate catabolism	GATCTATGGGG	-977	**38.3**	**25.4**	**1.5**
*INV*/β-fructofuranosidase [JX235361.1]	Carbohydrate catabolism	CCCCAGGCACT	-711	264.8	544.5	0.5
		TACAGATGGGG	-1,124			
		ATTAAATGGGG	-1,228			
*MLS1*/malate synthase [KX384892]	Glyoxylate cycle	ATTGGTTGGGG	-813	**305.8**	**137.8**	**2.2**
		GAATGGTGGGG	-1,605			
*RPO41*/mitochondrial RNA polymerase [KX384893]	Transcription from mitochondrial promoter	GATGACTGGGG	-169	54.3	45.5	1.2
*snf4*/Snf4, γ subunit of SNF1 kinase complex [KX384894]	Transcription regulation	AGTGGATGGGG	-340	**14.7**	**10.7**	**1.4**
		CCCCATAGAAC	-1,501			
*POX1*/Acyl CoA oxidase [KX384896]	Fatty acid β-oxidation	CCCCAGCCATC	-856	11.8	9.3	1.3
		CCCCCAAGAAAC	-1,063			
		AGAACTTGGGG	-1,252			
		TTGATCTGGGG	-1,342			
*grg2*/glucose repressible gene 2 [JN043364]	Unknown function	GCGGAC	-342	20,289.0	18,200.8	1.1
		GCGGAG	-214			
*ADH*/alcohol dehydrogenase [KX384885]	Alcohol metabolism	CCCCATCGCAA	-671	1,580.4	1,725.2	0.9
*PDC*/Pyruvate decarboxylase [HQ694557.1]	Alcohol metabolism	none	-	1,492.3	1,371.7	1.1
*ACT*/Actin [X89898.1]	Structural constituent of cytoskeleton	none	-	920.9	990.4	0.9

*Upstream position relative to the translation start codon. Ratio (mut/wt): ratio between the RPKM value of each transcript in the mutant and wild-type strain. Transcripts that have a higher RPKM value in the Xd*mig1*^-/-^ strain compared to the wild-type are highlighted in bold.

To search for other potential genes regulated by Mig1, transcriptomic data from both strains was analyzed to identify differentially expressed genes (DEGs) between the wild-type and *Xdmig1*^*-/-*^ strains cultured with glucose as the sole carbon source using the statistical programs EdgeR, EBSeq and DESeq2, as these statistical methods were validated in a previous study [62]. The three programs identified different numbers of DEGs; EBSeq was the least strict method, finding 1,567, while only 304 and 22 DEGs were found with EdgeR and DESeq2, respectively. Notably, the 22 DEGs identified with DESeq2 were all included among the 304 DEGs found with EdgeR. Among these 304 DEGs, 177 and 127 were overexpressed and underexpressed, respectively, in the *Xdmig1*^*-/-*^ strain compared to the wild-type. As Mig1 is a transcriptional repressor, it is possible that the underexpression of some transcripts in the *Xdmig1*^*-/-*^ strain is attributable to indirect effects; thus, only the 177 overexpressed transcripts may be direct Mig1 targets. When BLASTx analyses were performed, deduced proteins from only 45 of the 177 overexpressed DGEs demonstrated over 30% sequence identity with previously described proteins available in the NCBI database (S2 Table), while the remainder showed high identity percentages with proteins described as “hypothetical proteins” or, in some cases, did not match any of the available sequences. Conversely, in the RPKM value analysis, 665 transcripts exhibited at least a 5-fold higher RPKM value in the *Xdmig1*^*-/-*^ transcriptome compared to the wild-type; among them, 100 transcripts coincided with DEGs identified as overexpressed by EdgeR analysis, and 27 coincided with 45 proteins to which a possible function could be attributed according to BLASTx analysis. Thus, among the transcripts of genes that potentially encode functional proteins with known functions, 27 out of a total of 45 (S2 Table) were identified as overexpressed according to both criteria employed (RPKM value and EdgeR statistical analyses).

The 45 identified proteins included proteins involved in transmembrane transport, transcriptional regulation and isoprenoid metabolism (S2 Table). The majority of the overexpressed DEGs identified in the *Xdmig1*^*-/-*^ strain are involved in the transmembrane transport of different substrates, primarily those involved in ion homeostasis and in the uptake of nitrogen compounds. These results are not surprising because Mig1 is involved in the repression of several genes, including genes encoding transporters that are activated under stress conditions such as starvation or osmotic stress [2,63,64]. In addition, overexpressed DEGs included genes encoding proteins involved in isoprenoid metabolism; among them were the potential mevalonate kinase encoding gene, which would be involved in the mevalonate pathway that synthesizes carotenogenesis precursors in non-photosynthetic organisms such as yeasts. Thus, its overexpression could also contribute to the elevated carotenoid production observed in the *Xdmig1*^*-/-*^ strain. Transcriptomic analyses and the results from RT-qPCR experiments indicate that the gene product of the identified *X*. *dendrorhous MIG1* gene is involved in regulating the expression of genes involved in several biological processes, including carotenogenesis.

#### Effects of the *X*. *dendrorhous MIG1* gene mutation on extracellular invertase activity

**Fig 6:**
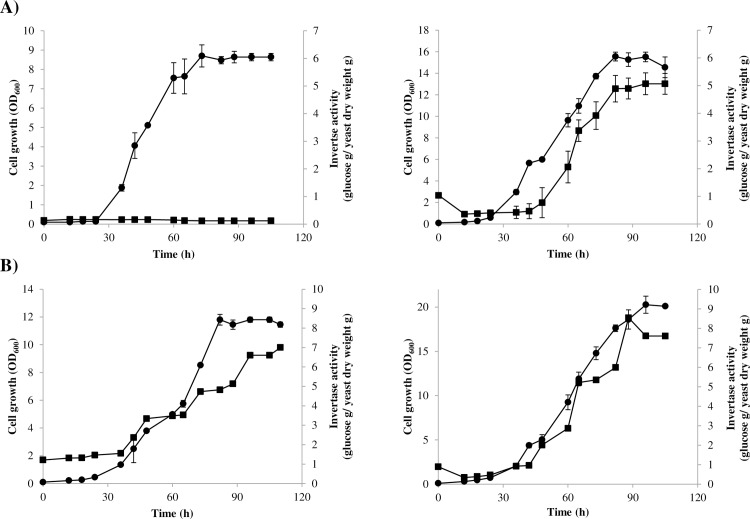
Growth curves and extracellular invertase activity in the wild-type and *Xdmig1*^*-/-*^ strains cultured with glucose or maltose as the sole carbon source. On the left, data from the wild-type; on the right, data from strain *Xdmig1*^*-/-*^. Strains were cultured at 22°C in MMv medium supplemented either with A) 2% glucose or B) 2% maltose. Yeast growth (circles) and invertase activity (squares) are represented. Values represent the average of three independent cultures, and error bars correspond to the standard deviation.

To further address the functional consequences of Mig1 inactivation in *X*. *dendrorhous*, extracellular invertase activity was evaluated in the wild-type and mutant strains (Fig 6). For this, both strains were cultured in MMv supplemented with glucose or maltose, and growth and invertase activity profiles were evaluated. As expected, invertase activity was detected and increased during growth of the wild-type strain only in maltose-based cultures because catabolic repression mediated by Mig1 is not functional under these conditions. However, this activity was detected in *Xdmig1*^*-/-*^ mutant strain cultures supplemented either with glucose or maltose. It is noteworthy that a potential Mig1 box was identified in the promoter of the *INV* gene, which is responsible for the invertase activity analyzed in this study (Table 3). These results demonstrate that catabolic repression of extracellular invertase activity is abolished in the *Xdmig1*^*-/-*^ mutant strain.

### Heterologous complementation of the *X*. *dendrorhous MIG1* gene in *S*. *cerevisiae*

The results obtained from the *Xdmig1*^*-/-*^ mutant strain strongly suggest that the identified *MIG1* gene product is indeed involved in catabolic repression and that this mechanism regulates, at least in part, carotenogenesis in *X*. *dendrorhous*. To confirm the functionality of the identified gene, heterologous complementation assays were performed in a *mig1*^*-*^ *S*. *cerevisiae* (*Scmig1*^-^) mutant strain. For this, plasmid YEpNP-MIG1 (Table 1) was constructed to express the *X*. *dendrorhous MIG1* gene in the *S*. *cerevisiae Scmig1*^-^ strain, giving rise to strain *Scmig1*^*-*^(YXd). Strain *Scmig1*^-^ was also transformed with the empty vector YEpNP as a control (strain *Scmig1*^*-*^(Y-)). To study gene complementation, a *S*. *cerevisiae* wild-type strain (S288c) was also included for comparative purposes.

First, the ability of strains S288c, *Scmig1*^*-*^(Y-) and *Scmig1*^*-*^(YXd) to grow using an alternative glucose in the presence of 2DG, which exerts catabolic repression, was evaluated. The three strains were able to grow with glucose as the sole carbon source in the presence of 2DG, but only strain *Scmig1*^*-*^(Y-), carrying the empty vector, was able to grow with raffinose or sucrose in the presence of the glucose analog. These results indicate that, only in this last strain, the extracellular invertase activity necessary to metabolize sucrose and raffinose is not repressed by the presence of 2DG, demonstrating that the *X*. *dendrorhous MIG1* gene complements the *mig1*^-^ mutation in *S*. *cerevisiae*. This complementation leads to the repression of the *S*. *cerevisiae* invertase encoding gene (*SUC2*), which is regulated by catabolic repression [13]. To confirm this observation, extracellular invertase activity after the strains were cultivated in SD medium with galactose supplementation, with or without glucose, was evaluated (Table 4).

**Table 4:** Invertase activity in *S*. *cerevisiae* strains cultured in galactose supplemented with or without glucose.

	Invertase activity (g of glucose released/g of dry yeast weight)		
Carbon Source	S288c	*Scmig1*^*-*^(Y-Xd)	*Scmig1*^*-*^(Y-)
**Gal 2%**	3.3 ± 0.5	1.30 ± 0.08	1.45 ± 0.02
**Gal 2% + Glu 2%**	0.5 ± 0.1*	0.45 ± 0.04*	1.42 ± 0.08

Extracellular invertase activity was evaluated. Gal: galactose; Glu: glucose. The average activity value from three independent cultures ± the standard deviation is indicated. Significant differences between Gal 2% and Gal 2% + Glu 2% cultures of the same strain are indicated (Student’s t test; *p ≤ 0.05).

The strain S288c exhibited the highest extracellular invertase activity value when cultured only in the presence of galactose; however, this activity decreased 6-fold when the strain was cultured with both carbon sources, indicating that the presence of glucose repressed this enzymatic activity, as expected. Strains *Scmig1*^*-*^(Y-Xd) and *Scmig1*^*-*^(Y-) demonstrated equivalent extracellular invertase activity levels when cultured with galactose, but when cultured in the presence of glucose, enzymatic activity was reduced by approximately 3-fold (Student’s t test, p ≤ 0.05) only in strain *Scmig1*^*-*^(Y-Xd), and no differences were observed between both conditions in strain *Scmig1*^*-*^(Y-). These results indicate that extracellular invertase activity in *S*. *cerevisiae* is repressed by glucose only in the presence of a functional *MIG1* gene, demonstrating that the identified *MIG1* gene from *X*. *dendrorhous* indeed complements the *S*. *cerevisiae mig1*^-^ mutation, at least for the repression of invertase activity.

## Conclusions

Catabolic repression is an operative process in *X*. *dendrorhous*. In this study, the *MIG1* gene from *X*. *dendrorhous* was characterized, and its involvement in catabolic repression was demonstrated by gene mutation in *X*. *dendrorhous* and by heterologous complementation in *S*. *cerevisiae*. In both yeast species, the presence of the identified gene affected the ability of the yeasts to utilize alternative carbon sources and contributed to invertase activity repression in the presence of glucose. The *mig1*^-^ mutation partially releases the glucose-mediated repression of carotenoid biosynthesis in *X*. *dendrorhous*, supporting the notion that carotenogenesis is regulated, at least in part, by catabolic repression in this yeast. This new finding will contribute to the design of innovative genetic strategies to enhance carotenoid production in this biotechnologically relevant microorganism.

## Supporting Information

S1 Fig Mig1 EMSA assays. Mig1 binding to DNA was evaluated by employing DNA fragments, approximately 200 bp in length, of the promoter regions of the *X*. *dendrorhous* carotenogenic genes A) *crtI* (primers PcrtI.1000.Fw + crtI.Mig1.Rv: 249 bp) and B) *crtYB* (primers PcrtYB.1000.Fw + crtYB.Mig1.Rv: 254 bp), as well as C) three fragments from the promoter region of the *crtS* gene containing the first (box A, primers crtS1000.Fw + crtS.Mig1-854.Rv: 183 bp), the second and third (boxes B and C, primers PS1Fw + PS1Rv: 204 bp), and the third and fourth (boxes C and D, primers PS2Fw + PS2Rv: 209 bp) Mig1 boxes that were bioinformatically identified. Labeled DNA was incubated with non-specific cold DNA and the purified *X*. *dendrorhous* Mig1 protein before electrophoresis; lane (a): labeled DNA without protein incubation (control), lane (b): labeled DNA previously incubated with Mig1.
(TIF)

S2 Fig Growth curves of the wild-type and *Xdmig1*^*-/-*^ mutant strains. Strains were cultured at 22°C with constant agitation in A) YM- 1% glucose medium and in B) MMv medium supplemented with 2% glucose. Data from the wild-type strain (triangles) and *Xdmig1*^*-/-*^ strain (squares) are represented. Values correspond to the average of three independent cultures, and error bars indicate the standard deviation.
(PDF)

S1 Table Primers used in this work. (DOCX)

S2 Table Identified overexpressed DEGs in strain *Xdmig1*^*-/-*^ using EdgeR and BLASTx analysis. (DOCX)
