# Supplementary figures and images for: The Involvement of Mig1 from Xanthophyllomyces dendrorhous in Catabolic Repression: An Active Mechanism Contributing to the Regulation of Carotenoid Production
Source: PLoS One. 2016 Sep 13;11(9):e0162838. doi: 10.1371/journal.pone.0162838 (PMC5021340; doi:10.1371/journal.pone.0162838)

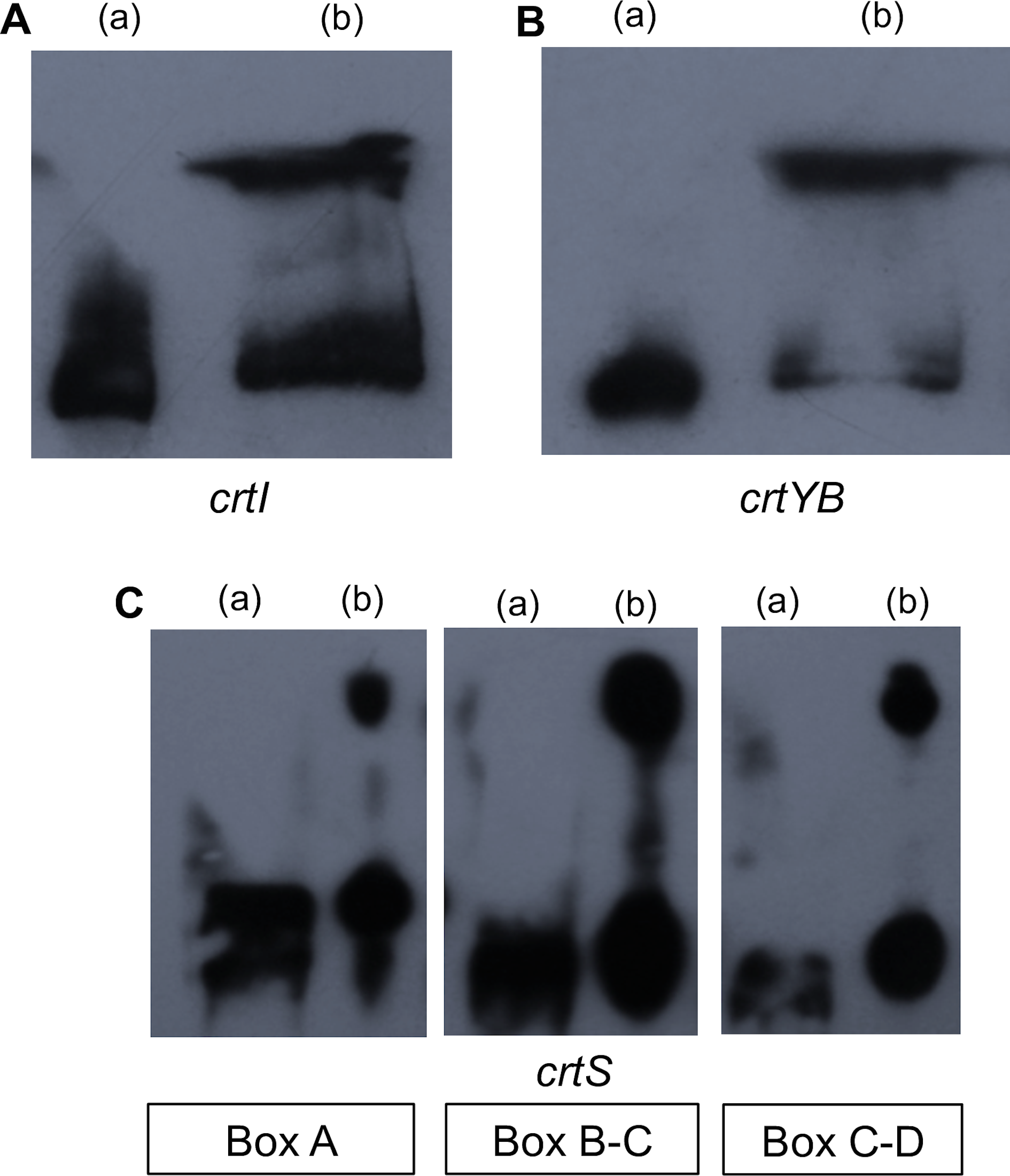

Supplement: S1 Fig — Mig1 binding to DNA was evaluated by employing DNA fragments, approximately 200 bp in length, of the promoter regions of the X. dendrorhous carotenogenic genes A) crtI (primers PcrtI.1000.Fw + crtI.Mig1.Rv: 249 bp) and B) crtYB (primers PcrtYB.1000.Fw + crtYB.Mig1.Rv: 254 bp), as well as C) three fragments from the promoter region of the crtS gene containing the first (box A, primers crtS1000.Fw + crtS.Mig1-854.Rv: 183 bp), the second and third (boxes B and C, primers PS1Fw + PS1Rv: 204 bp), and the third and fourth (boxes C and D, primers PS2Fw + PS2Rv: 209 bp) Mig1 boxes that were bioinformatically identified. Labeled DNA was incubated with non-specific cold DNA and the purified X. dendrorhous Mig1 protein before electrophoresis; lane (a): labeled DNA without protein incubation (control), lane (b): labeled DNA previously incubated with Mig1. (TIF) [file pone.0162838.s001.tif]

**A)**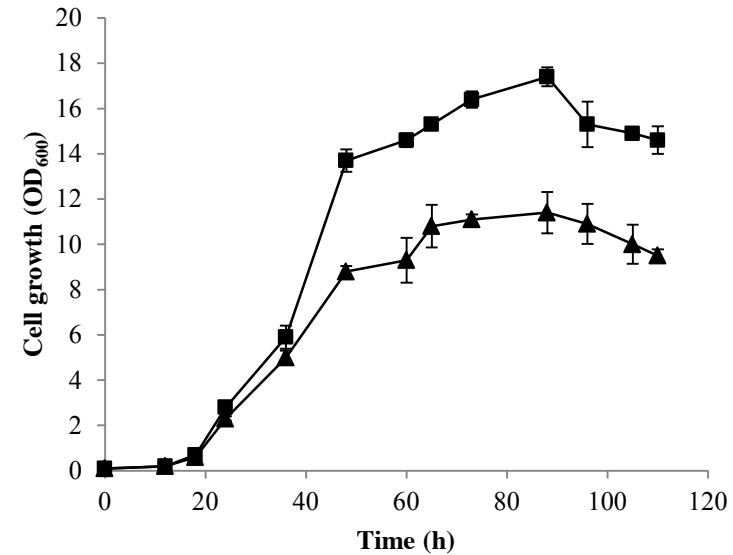**B)**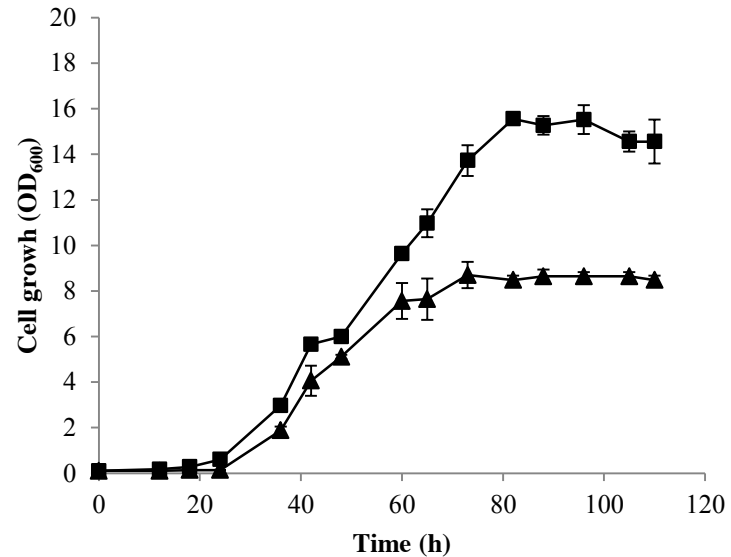

Supplement: S2 Fig — Strains were cultured at 22°C with constant agitation in A) YM- 1% glucose medium and in B) MMv medium supplemented with 2% glucose. Data from the wild-type strain (triangles) and Xdmig1-/- strain (squares) are represented. Values correspond to the average of three independent cultures, and error bars indicate the standard deviation. (PDF) [file pone.0162838.s002.pdf]
