# Supplementary material for: The Involvement of Mig1 from Xanthophyllomyces dendrorhous in Catabolic Repression: An Active Mechanism Contributing to the Regulation of Carotenoid Production
Source: PLoS One. 2016 Sep 13;11(9):e0162838. doi: 10.1371/journal.pone.0162838 (PMC5021340; doi:10.1371/journal.pone.0162838)
Supplement: S1 Table — (DOCX) [file pone.0162838.s003.docx]

**S1 Table. Primers used in this work.**

| **Primer name** | **Hybridization target [GenBank] / Objective for design** | **5’ to 3’ Sequence (Orientation)** |
| --- | --- | --- |
| ***Primers used for MIG1 cDNA synthesis:*** | | |
| Mig1cDNA | *X. dendrorhous* *MIG1* [KX384897]. | atgtcaacattcttcaacgaca (F) |
| Mig1cDNA_Stop | *X. dendrorhous* *MIG1* [KX384897]. | ttagctaaccattgcgtctgaa (R) |
| Mig1_Fw1480 | *X. dendrorhous* *MIG1* [KX384897]. | tggtggaatggataccgatc (F) |
| Mig1cDNARV2 | *X. dendrorhous* *MIG1* [KX384897]. | ttgtgacgaggtcgtgtttatg (R) |
| ***Primers used to construct and evaluate the Xdmig1^-/-^  mutant strain:*** | | |
| Mig1up2.F | “up” *MIG1* Fragment. | tacactcaaaactcacctcg (F) |
| 10mig.up2-50TEF.R | “up” *MIG1* Fragment. | **gacgatctaggcaaagagcttgtgtcggatgaactgtcggctgatgagcc**gcctgggaat (R) |
| 10mig.up2-50TEF.R | Hygromycin B resistance module. | **tacagtcacagaggccgcgttgacgatggacaagagcaaaattcccaggc**ggctcatcag (F) |
| gpdT-50IIMig1dw.R | Hygromycin B resistance module. | **gtgacgcatctcgtattctgctcgacggacagactcagaacgctccatct**atcatgagag (R) |
| 50.IIMig1.dw-10gpd.T.F | “down” *MIG1* Fragment. | **ttcaatcacatctgttgaccatcaccatcatctccgtcatctctcatgat**agatggagcg (F) |
| IIMig1.dw.R | “down” *MIG1* Fragment. | gaagcggttttcgatagctc (R) |
| out.Mig1up.F | To evaluate the resistance module integration at the *MIG1* locus*.* | gttgtctggtggttctcatc (F) |
| out.Mig1dw.R | To evaluate the resistance module integration at the *MIG1* locus*.* | tgaatggtgagagccctctg (R) |
| ***Primers used in RT-qPCR analyses:*** | | |
| mactF-RT | *X. dendrorhous* *act* [X89898.1]. | ccgccctcgtgattgataac (F) |
| mactR-RT | *X. dendrorhous* *act* [X89898.1]. | tcaccaacgtaggagtcctt (R) |
| mmcrtYBF2-RT | *X. dendrorhous* *crtYB* [DQ016503] . | tcgcatattaccagatccatctga (F) |
| mmcrtYBR2-RT | *X. dendrorhous* *crtYB* [DQ016503]. | ggatatgtccatgcgccatt (R) |
| mmcrtIF-RT | *X. dendrorhous* *crtI* [DQ028748.1]. | catcgtgggatgtggtatcg (F) |
| mmcrtIR-RT | *X. dendrorhous* *crtI* [DQ028748.1]. | ggcccctgatcgaatcgataa (R) |
| mcrtSF-RT | *X. dendrorhous crtS* [DQ201828.1]. | atggctcttgcagggtttga (F) |
| mcrtSR-RT | *X. dendrorhous crtS* [DQ201828.1]. | tgctccataagctcgatcccaa (R) |
| grg2real FW1 | *X. dendrorhous* *grg2* [JN043364]. | catcaagacctctgtcaccaac (F) |
| grg2real RV1 | *X. dendrorhous* *grg2* [JN043364]. | ttggcgtcagacgaggact (R) |
| pdcreal FW1 | *X. dendrorhous* *PDC* [HQ694557.1]. | tcaacactgagctgcccact (F) |
| pdcreal RV1 | *X. dendrorhous* *PDC* [HQ694557.1]. | attccgaatcgggaagcaca (R) |
| ***Primers used for EMSA assays:*** | | |
| crtS1000.Fw | *X. dendrorhous crtS* gene promoter containing Box A. | ggcatcgaaacctgggctgac (F) |
| crtS.Mig1-854 Rv | *X. dendrorhous crtS* gene promoter containing Box A. | gccacagcggcctcagtggcagcacttgctccagacgcagttgcagggcctaaagcc (R) |
| PS1Fw | *X. dendrorhous crtS* gene promoter containing Boxes B-C. | catccatcagaggtgcagcc (F) |
| PS1Rv | *X. dendrorhous crtS* gene promoter containing Boxes B-C. | cattcaaccggtcgaatagg (R) |
| PS2Fw | *X. dendrorhous crtS* gene promoter containing Boxes C-D. | gaagatgatgcggcggcaac (F) |
| PS2Rv | *X. dendrorhous crtS* gene promoter containing Boxes C-D. | gatgcatcacctgctgctc (R) |
| PcrtI.1000.Fw | *X. dendrorhous* *crtI* gene promoter. | ctttcctccttctcttccattc (F) |
| crtI.Mig1.Rv | *X. dendrorhous* *crtI* gene promoter. | gttgaataaatccaaagactaacctgtatctgtggggcttcttgacacctccagcag (R) |
| PcrtYB.1000.Fw | *X. dendrorhous* *crtYB* gene promoter. | cagaagatgggtccaccgatagtgg (F) |
| crtYB.Mig1.Rv | *X. dendrorhous* *crtYB* gene promoter. | cgaggtaatgagctgaagtagaagctaatgattttggggatcgcccaaccaccatagttc (R) |
| grg2.1-348.Fw | *X. dendrorhous* *grg2* gene promoter containing Box A. | gaggtatatgcgttccgcgaactgcggagatgtgacgtcacatctcgtcacagc (F) |
| grg2.1-348.Rv | *X. dendrorhous* *grg2* gene promoter containing Box A. | gctgtgacgagatgtgacgtcacatctccgcagttcgcggaacgcatatacctc (R) |
| grg2.1-220.Fw | *X. dendrorhous* *grg2* gene promoter containing Box B. | gaccgggctcgactgtcgaacgagcggaccagagatgccggcgagatgggctg (F) |
| grg2.1-220.Rv | *X. dendrorhous* *grg2* gene promoter containing Box B. | cagcccatctcgccggcatctctggtccgctcgttcgacagtcgagcccggtc (R) |

F: forward; R: reverse. 5’ complementary ends of primers used to construct the *Xdmig1^-/-^* strain are highlighted in bold. (F) and (R): indicates primer orientation, forward or reverse.
