# Supplementary material for: The Involvement of Mig1 from Xanthophyllomyces dendrorhous in Catabolic Repression: An Active Mechanism Contributing to the Regulation of Carotenoid Production
Source: PLoS One. 2016 Sep 13;11(9):e0162838. doi: 10.1371/journal.pone.0162838 (PMC5021340; doi:10.1371/journal.pone.0162838)
Supplement: S2 Table — (DOCX) [file pone.0162838.s004.docx]

**S2 Table. Identified overexpressed DEGs in strain *Xdmig1^-/-^* using EdgeR and BLASTx analysis.**

|  | **BLAST Results** | | | | |
| --- | --- | --- | --- | --- | --- |
| **GenBank Nº** | **Potential Gene Product** | **Biological Process** | **Organism** | **E-value** | **Identity (%)** |
| **[KX384932]** | **Uridine permease/thiamine transporter** | **Transmembrane transport** | ***Rhizoctonia solani*** | **1.8 E-166** | **47** |
| [KX384899] | MFS general substrate transporter | Transmembrane transport | *Rhodosporidium toruloides* | 2.3 E-143 | 59 |
| **[KX384912]** | **Potassium transporter** | **Transmembrane transport/cellular potassium ion homeostasis** | ***Stereum hirsutum*** | **3.9 E-130** | **42** |
| **[KX384942]** | **Copper transporter, low affinity** | **Transmembrane transport/copper ion import** | ***Trametes versicolor*** | **1.0 E-32** | **43** |
| **[KX384930]** | **Siderophore-iron transporter Str1** | **Transmembrane transport/ iron ion homeostasis** | ***Cryptococcus gattii*** | **4.0 E-95** | **70** |
| **[KX384923]** | **MFS general substrate transporter** | **Transmembrane transport** | ***Colletotrichum gloeosporioides*** | **1.5 E-24** | **53** |
| **[KX384922]** | **Cation transport ATPase/ copper resistance associated** | **Transmembrane transport/cellular metal ion homeostasis** | ***Rhizoctonia solani*** | **0.00** | **40** |
| **[KX384898]** | **Xanthine uracil permease** | **Transmembrane transport of organic acids** | ***Trametes versicolor*** | **0.00** | **70** |
| **[KX384914]** | **Aminoacid transporter/ high affinity methionine permease** | **Aminoacid transmembrane transport** | ***Coniophora puteana*** | **0.00** | **64** |
| **[KX384902]** | **Urea transporter** | **Urea transport and catabolic process** | ***Laccaria bicolor*** | **0.00** | **60** |
| **[KX384907]** | **Ammonium transporter** | **Ammonium transmembrane transport/ nitrogen utilization** | ***Punctularia strigosozonata*** | **0.00** | **73** |
| [KX384904] | Carbon-nitrogen hydrolase /nitrylase | Nitrogen compound metabolism | *Auricularia delicata* | 6.3 E-75 | 53 |
| **[KX384921]** | **Guanine deaminase** | **Guanine catabolic process** | ***Cylindrobasidium torrendii*** | **3.5 E-86** | **48** |
| **[KX384918]** | **Phosphoribosylaminoimidazolecarboxamide formyltransferase** | **Purine biosynthesis** | ***Cryptococcus gattii*** | **0.00** | **79** |
| **[KX384906]** | **Urate oxidase** | **Purine metabolism** | ***Cryptococcus gattii*** | **1.8 E-102** | **55** |
| [KX384917] | Clavaminate synthase like protein/α-ketoglutarate-dependent xanthine dioxygenase | Xanthine metabolism | *Rhizoctonia solani* | 0.00 | 66 |
| [KX384903] | Integrase | DNA integration/recombination | *Rozella allomycis* | 2.1 E-22 | 45 |
| **[KX384929]** | **DNA mismatch repair protein/MSH5** | **DNA repair/ mismatch repair** | ***Coprinopsis cinerea*** | **1.4 E-7** | **31** |
| [KX384908] | Glutathione-S-transferase | Glutathione metabolic process/ cell detoxification | *Ustilago maydis* | 1.6 E-90 | 58 |
| [KX384934] | FAD dependent pyridine nucleotide-disulphide oxidoreductase | Glutathione metabolic process/ cell redox homeostasis | *Penicillium expansum* | 6.6 E-17 | 35 |
| [KX384926] | NADH dehydrogenase (ubiquinone) | Cell redox homeostasis | *Ceraceosorus bombacis* | 2.0 E-42 | 40 |
| **[KX384939]** | **Ferric reductase** | **Iron ion homeostasis** | ***Cryptococcus neoformans*** | **2.4 E-81** | **33** |
| [KX384933] | GSP1/Ras superfamily | Intracellular protein transport/signal transduction | *Melanopsichium pennsylvanicum* | 8.2 E-77 | 61 |
| [KX384919] | GPI-inositol deacylase | Intracellular protein transport | *Moniliophtora roreri* | 1.72 E-99 | 30 |
| [KX384931] | Membrane coat complex retromer, subunit VPS5/SNX1 | Intracellular protein transport | *Trametes versicolor* | 1.9 E-102 | 51 |
| **[KX384916]** | **Biotin-[acetyl-CoA-carboxylase] ligase** | **Protein modification** | ***Cryptococcus gattii*** | **2.8 E-155** | **39** |
| [KX384941] | Phosphoglycerate dehydrogenase | Amino acid biosynthetic process | *Cryptococcus gattii* | 6.07 E-57 | 47 |
| [KX384901] | PLP-dependent aminotransferase | Cellular amino acid metabolic process | *Fomitiporia mediterranea* | 1.5 E-109 | 43 |
| **[KX384900]** | **Translation factor YwlC** | **Translation** | ***Postia placenta*** | **3.1 E-69** | **45** |
| [KX384909] | G1/S specific cyclin Pcl1 | Cell cycle | *Cryptococcus neoformans* | 4.0 E-61 | 49 |
| **[KX384915]** | **Mak16 protein** | **Cell cycle/ ribosome biogenesis** | ***Trametes versicolor*** | **6.4 E-116** | **85** |
| **[KX384938]** | **U3 small nucleolar ribonucleoprotein LCP5** | **rRNA processing/ ribosome biogenesis** | ***Cryptococcus gattii*** | **3.3 E-12** | **34** |
| **[KX384905]** | **Rrp9/WD40 repeat protein** | **rRNA processing** | ***Rhodosporidium toruloides*** | **4,3 E-133** | **44** |
| **[KX384937]** | **Sfp1, zinc finger protein** | **Transcription regulation/positive regulation of ribosomal protein gene transcription** | ***Rhizoctonia solani*** | **1.5 E-22** | **49** |
| [KX384935] | Transcriptional repressor/CCR4-NOT transcriptional complex, subunit 4 | Transcription regulation/mRNA catabolism | *Rhizoctonia solani* | 4.5 E-86 | 56 |
| [KX384911] | DNA directed RNA polymerase III subunit Rpc4 | tRNA transcription from RNA polymerase III promoter | *Rhizoctonia solani* | 3.5 E-7 | 43 |
| **[KX384910]** | **Alpha -beta hydrolase/ Lysophospholipase** | **Lipid metabolism** | ***Heterobasidion irregulare*** | **5.1 E-81** | **42** |
| [KX384940] | Phytanoil-CoA dioxygenase | Lipid metabolism | *Aspergillus kawachii* | 1.55 E-93 | 46 |
| [KX384925] | Short chain dehydrogenase reductase SDR | Alcohol metabolism process/sterol metabolic process | *Penicillium solitum* | 3.3 E-50 | 37 |
| **[KX384928]** | **3 beta hydroxysteroid dehydrogenase isomerase** | **Sterol biosynthetic process** | ***Gloeophyllum trabeum*** | **1.7 E-47** | **33** |
| **[KX384927]** | **Mevalonate kinase** | **Sterol/isoprenoid biosynthesis** | ***Cryptococcus neoformans*** | **2.1 E-74** | **47** |
| **[KX384920]** | **2-dehydropantoate 2-reductase/ 6 phosphogluconate dehydrogenase C-terminal domain like protein** | **pantoate byosynthetic process** | ***Rhizoctonia solani*** | **1.0 E-55** | **37** |
| **[KX384913]** | **Glycosyl transferase family 8 protein** | **Carbohidrates metabolism** | ***Botryobasidium botryosum*** | **4.2 E-105** | **50** |
| [KX384924] | NDUFA5 subunit of NADH ubiquinone reductase | Respiratory electron transport chain | *Moniliophtora roreri* | 6.6 E-34 | 51 |
| **[KX384936]** | **GPI anchored protein** | **Cell wall organization** | ***Rhodosporidium toruloides*** | **5.1 E-36** | **50** |

Transcripts having a RPKM value at least 5-fold higher in strain *Xdmig1^-/-^* compared to the wild-type are highlighted in bold. GPI: Glycophosphatidylinositol, CoA: co-enzyme A, MFS: Major Facilitator Superfamily.
